# Structures of parasite calreticulins provide insights into their flexibility and dual carbohydrate/peptide-binding properties

**DOI:** 10.1107/S2052252516012847

**Published:** 2016-09-14

**Authors:** Christophe Moreau, Gianluca Cioci, Marina Iannello, Emmanuelle Laffly, Anne Chouquet, Arturo Ferreira, Nicole M. Thielens, Christine Gaboriaud

**Affiliations:** a Institut de Biologie Structurale (IBS), Université Grenoble Alpes, CEA, CNRS, 38044 Grenoble, France; b Program of Immunology, Institute of Biomedical Sciences (ICBM), Faculty of Medicine, University of Chile, Santiago, Chile

**Keywords:** protein structure, molecular recognition, X-ray crystallography, solution scattering, calreticulin, parasites

## Abstract

Calreticulin (CRT) is a multifaceted protein, initially discovered as an endoplasmic reticulum (ER) chaperone protein, that is essential in calcium metabolism. Various implications in cancer, early development and immunology have been discovered more recently for CRT, as well as its role as a dominant ‘eat-me’ prophagocytic signal. Intriguingly, cell-surface exposure/secretion of CRT is among the infective strategies used by parasites such as *Trypanosoma cruzi*, *Entamoeba histolytica*, *Taenia solium*, *Leishmania donovani* and *Schistosoma mansoni*. Because of the inherent flexibility of CRTs, their analysis by X-ray crystallography requires the design of recombinant constructs suitable for crystallization, and thus only the structures of two very similar mammalian CRT lectin domains are known. With the X-ray structures of two distant parasite CRTs, insights into species structural determinants that might be harnessed to fight against the parasites without affecting the functions of the host CRT are now provided. Moreover, although the hypothesis that CRT can exhibit both open and closed conformations has been proposed in relation to its chaperone function, only the open conformation has so far been observed in crystal structures. The first evidence is now provided of a complex conformational transition with the junction reoriented towards P-domain closure. SAXS experiments also provided additional information about the flexibility of *T. cruzi* CRT in solution, thus complementing crystallographic data on the open conformation. Finally, regarding the conserved lectin-domain structure and chaperone function, evidence is provided of its dual carbohydrate/protein specificity and a new scheme is proposed to interpret such unusual substrate-binding properties. These fascinating features are fully consistent with previous experimental observations, as discussed considering the broad spectrum of CRT sequence conservations and differences.

## Abstract 2

New insights into calreticulins are obtained by studying parasite species and combining a dissection strategy, X-ray crystallography and SAXS.

## 1. Introduction   

Calreticulin (CRT) is a 46 kDa soluble multifunctional protein that was initially identified as an endoplasmic reticulum (ER) chaperone for monoglycosylated proteins and is also involved in Ca^2+^ regulation. Since the ER plays a central role in the protein-secretion pathway, it contains many protein chaperones and high-level checkpoints for post-translational modifications and folding-quality control (Braakman & Bulleid, 2011 ▸). Similar to its ER membrane homologue calnexin (CNX) ectodomain, CRT is composed of a globular domain, into which a flexible arm-like P (proline-rich) domain is inserted (Schrag *et al.*, 2001 ▸). In addition, a highly charged and flexible C-terminus extension in CRT and CNX can bind numerous calcium ions with low affinity (Michalak *et al.*, 2002 ▸; Wijeyesakere *et al.*, 2016 ▸). The globular domain is made up of seven concave and six convex β-sheets forming a legume lectin fold (Chouquet *et al.*, 2011 ▸; Kozlov *et al.*, 2010 ▸; Schrag *et al.*, 2001 ▸). It contains the lectin site, which specifically recognizes the tetrasaccharide Glc_1_Man_3_ (G1M3), stabilizing the glucose moiety through amino acids Gly124, Tyr128, Met131, Ile147, Lys111 and Asn154 in murine CRT (MmCRT; Kozlov *et al.*, 2010 ▸). This glycan-dependent binding mainly defines the *in vivo* interactions of CRT in the ER. However, CRT can also bind nonglycosylated proteins *in vivo* and prevent their aggregation *in vitro* (Pocanschi *et al.*, 2011 ▸). When exported at the cell surface, CRT can bind several targets that are involved in a wide variety of functions (Gold *et al.*, 2010 ▸; de Bruyn *et al.*, 2015 ▸). For example, it can be a very potent phagocytosis ‘eat-me’ signal (Gardai *et al.*, 2005 ▸; Feng *et al.*, 2015 ▸).

CRT also appears to be highly conserved in several parasite species from protists (*Trypanosoma*, *Amoeba* and *Leishmania*) to helminths (*Onchocerca*, *Schistosoma* and *Taenia*), and it was suggested early on that it could play an important role in their biology, for example in the adaptation of the parasite to various environments and in escape from host immune responses (Nakhasi *et al.*, 1998 ▸; Ferreira *et al.*, 2004 ▸). We have investigated the CRTs of two distinct parasites, *Trypanosoma cruzi* (TcCRT) and *Entamoeba histolytica* (EhCRT), for which a pathogenic role has been proposed. These two protist parasites are phylogenetically very distant from mammals and they do not have, for example, any protein analogue of CNX. *T. cruzi* is the causative agent of Chagas disease, an acute illness affecting 12 million people in Latin America and causing 50 000 deaths per year. Cell-surface expression of TcCRT only occurs in its infective trypomastigote stage (Sosoniuk *et al.*, 2014 ▸), where it has been proposed to enhance host-cell infection (Ramírez *et al.*, 2011 ▸) and mother-to-child transmission (Castillo *et al.*, 2013 ▸). *E. histolytica* is the causative agent of amoebiasis, a disease mainly characterized by dysentery that leads to about 100 000 deaths per year. Dysentery occurs after the invasion of the colonic epithelium. This invasive form of the disease is characterized by the amoebic phagocytosis of human immune cells and erythrocytes. During this process, EhCRT is exported from the ER to the phagolysosomal cupule, where it favours the phagocytosis process in a way similar to that described for human CRT on the surface of macrophages during the clearance of apoptotic cells (Vaithilingam *et al.*, 2012 ▸; González *et al.*, 2011 ▸).

In this work, we present the X-ray structures of TcCRT and EhCRT, two parasite CRTs. A first example of a closed conformer is observed with EhCRT, and SAXS experiments conducted on TcCRT yielded new complementary insights into the dynamics of the flexible domains of CRT in solution. Besides, comparison of the phylogenetically distant parasite CRTs with their mammalian counterparts highlights key features involved in their common structure and chaperone function. Importantly, this study further supports the notion that the lectin site features dual substrate-binding properties, being able to bind both carbohydrates and/or proteins, and allows us to delineate two major subsites related to this dual binding property. Finally, these structures reveal species structural determinants which could be exploited to target the parasite CRTs without affecting the functions of the human host CRT.

## 2. Materials and methods   

### 2.1. Protein mutagenesis, production and purification   

Two genes optimized for *Escherichia coli* expression were synthesized and cloned into the pJexpress 411 vector (DNA2.0). Their sequences correspond to strain CL Brener for TcCRT (Q4CPZ0 in the UniProt sequence database; El-Sayed *et al.*, 2005 ▸) and strain HM-1:IMSS for EhCRT (UniProt reference F2VN92; González *et al.*, 2011 ▸). These two proteins were produced with a C-terminal 6×His tag. A large fragment of the P domain, namely residues 209–293 (TcCRT) and 204–288 (EhCRT), was replaced by a GSG linker in order to obtain constructs suitable for crystallization. This was performed using the following primers: 5′-CCGCGTGAGATTGTGGATGGCAGCGGTATCCCGAACCGGATTTT-3′ (TcCRT) and 5′-CCGAAAGAAATTGACGATGGCAG­CGGTATTGCGAACCCTGACTAC-3′ (EhCRT) and the corresponding reverse primers. The sequence used for EhCRT was initially truncated after Asn360. To introduce a C-terminal truncation after Lys368 in TcCRT, the following primers were used: 5′-GGAGGATATGGAAAAAGGCGACCACCATCACC-3′ (TcCRT) and the corresponding reverse primer. Mutagenic oligonucleotides were purchased from Eurogentec (Seraing, Belgium). The expression plasmids were generated using the QuikChange II XL Site-Directed Mutagenesis Kit (Agilent Technologies, Massy, France) according to the manufacturer’s protocol. The sequences of all constructs were verified by dsDNA sequencing (GATC Biotech, Mulhouse, France).

The TcCRT, EhCRT and HsCRT D71K mutant proteins were purified using a protocol highly similar to that described for HsCRT (Chouquet *et al.*, 2011 ▸). Briefly, transformed *E. coli* BL21 (DE3) cells were grown at 37°C, cooled for 1 h at 4°C and protein production was then induced by 1 m*M* IPTG for 20 h at 20°C. The cells were lysed in 50 m*M* Tris–HCl, 0.3 *M* NaCl, 5 m*M* CaCl_2_, 10 m*M* imidazole, 10% glycerol pH 7.5. The same buffer was used for the Ni^2+^ affinity chromatography purification step (HisTrap FastFlow, GE Healthcare Life Sciences). CRT-containing fractions were further purified on a Superdex 75 16/60 prep-grade gel-filtration column run in 20 m*M* Tris–HCl, 150 m*M* NaCl, 5 m*M* CaCl_2_ pH 8.0. Fractions containing the pure protein, as checked by SDS–PAGE, were concentrated to 12 mg ml^−1^ (TcCRT), 11 mg ml^−1^ (EhCRT) or 9.5 mg ml^−1^ (HsCRT D71K). These concentrations were determined by measuring the absorbance at 280 nm as computed from their amino-acid sequence.

### 2.2. Crystallization conditions   

Crystals were grown at 20°C using a hanging-drop vapour-diffusion setup. Very large but thin transparent TcCRT crystal plates were initially obtained with a reservoir solution consisting of 35% PEG 4000, 0.2 *M* sodium acetate, 0.1 *M* Tris–HCl pH 8.5. Their diffraction limit was about 2.9 Å. The resolution limit was increased to about 2.45 Å when 1 µl of 1 *M* glucose was initially added to 1 µl TcCRT and 1 µl of a reservoir solution consisting of 40% PEG 4000, 0.2 *M* sodium acetate, 0.1 *M* Tris–HCl pH 8.5. Two different EhCRT crystal forms were routinely obtained using 2.6–2.7 *M* ammonium sulfate as a precipitant in 0.1 *M* trisodium citrate buffer pH 5.0. HsCRT mutant crystals were obtained with a reservoir solution consisting of 30% PEG 4000, 0.2 *M* MgCl_2_, 0.1 *M* Tris–HCl pH 8.5.

### 2.3. X-ray diffraction data collection, structure determination and refinement   

The crystal diffraction data sets were collected on the beamlines of the European Synchrotron Radiation Facility (ESRF) in Grenoble (Table 1 ▸). The data were integrated with *XDS* (Kabsch, 2010 ▸) for TcCRT and using automated data integration for EhCRT (Monaco *et al.*, 2013 ▸). The data-collection statistics are listed in Table 1 ▸. The best data set for TcCRT was processed at 2.45 Å resolution. In this crystal form, six independent TcCRT monomers pack in space group *P*1, with unit-cell parameters *a* = 79.3, *b* = 79.4, *c* = 85.1 Å, α = 95.6, β = 98.7, γ = 119.9°. In the first EhCRT crystal form, three molecules are packed in space group *P*2_1_2_1_2_1_, with unit-cell parameters *a* = 74.4, *b* = 143.4, *c* = 171.6 Å. This crystal form allowed us to record a data set to 2.15 Å resolution (Table 1 ▸). The initial EhCRT crystal form allowed us to collect an accurate and redundant data set to 2.9 Å resolution. This second crystal form packs in space group *P*4_2_2_1_2, with unit-cell parameters *a* = 149.3, *b* = 149.3, *c* = 117.0 Å. The asymmetric unit contains two molecules.

The structures were solved by molecular replacement using *Phaser* (McCoy *et al.*, 2007 ▸). The native HsCRT structure (PDB entry 3pow; Chouquet *et al.*, 2011 ▸) was used as a starting model. Ten independent molecules were oriented in the crystal of the HsCRT D71K mutant protein. The truncated P fragment of the parasite CRTs was manually built into the electron density after several iterative refinement steps. The resulting models were extended manually with the help of *Coot* (Emsley *et al.*, 2010 ▸) and were improved by several cycles of refinement using *REFMAC* (Murshudov *et al.*, 2011 ▸) and *phenix.refine* (Adams *et al.*, 2010 ▸), including TLS refinements. The unknown positive density in the *F*_o_ − *F*_c_ difference map was investigated using *phenix.ligand_identification* (Adams *et al.*, 2010 ▸). The PDB Validation Server and *MolProbity* (Chen *et al.*, 2010 ▸) were used to check the quality of the model.

### 2.4. Small-angle X-ray scattering (SAXS)   

#### 2.4.1. Data collection and processing   

Data were collected on the BM29 beamline at the ESRF, Grenoble using a PILATUS 1M pixel detector (Dectris, Switzerland), an X-ray energy of 12.5 keV and beam dimensions of 0.7 × 0.7 mm. The distance between the detector and the sample was 2.867 m, covering a *q*-range of 0.025–5 nm^−1^. The *ATSAS* package was used to integrate and interprete the data (Petoukhov *et al.*, 2012 ▸). Full-length TcCRT and its crystallized construct were diluted with 20 m*M* Tris–HCl pH 8, 150 m*M* NaCl, 5 m*M* CaCl_2_ buffer to obtain four sample concentrations (Table 2 ▸). For each data set, 50 µl sample was injected into the flow and ten measurements of 1 s exposure were collected at 4°C. The first steps of data processing were performed automatically, including (i) removal of the diffusion scattering recorded for the buffer, (ii) averaging of ten frames and (iii) computing *I*(0) forward scattering. Optimized scattering curves were obtained using *PRIMUS* by merging the Guinier region of the data collected at 1.5 mg ml^−1^ (range 1–105), the Porod region of the data collected at 7 mg ml^−1^ (range 106–335) and the high-*q* region of the data collected at 10 mg ml^−1^ (range 336–1036) for the full-length TcCRT and the data collected at 1.8 mg ml^−1^ (range 1–605) and at 11 mg ml^−1^ (605–1036) for the TcCRT crystallized construct. The radius of gyration (*R*_g_) and the longest interatom distance (*D*_max_) were then calculated using *PRIMUS* and *GNOM* (Petoukhov *et al.*, 2012 ▸; Table 2 ▸).

#### 2.4.2. Rigid-body modelling   

Rigid-body modelling was performed using *BUNCH* (Petoukhov *et al.*, 2012 ▸) with default settings for an extended loop penalty weight (100), angular step (20°), initial annealing temperature (10°C), annealing steps (100) and successes to continue (100) and the data range *s* < 6 nm (crystallized construct) or *s* < 3nm (full length). The X-ray structure of TcCRT or the X-ray structure and modelled P arm and C-terminal end (initially modelled using the *Phyre*2 server; Kelley *et al.*, 2015 ▸) were used as input models. The calculation of theoretical scattering profiles of atomic structures and their fits to the experimental data were performed with *CRYSOL* (Petoukhov *et al.*, 2012 ▸). The ten best models were selected based on the χ^2^ values calculated by *CRYSOL* with high limits of 5 for full-length TcCRT and 1.2 for the crystallized construct. Finally, the models were compared by calculation of the normalized spatial discrepancy (NSD) using *DAMSEL* (Petoukhov *et al.*, 2012 ▸).

## 3. Results   

### 3.1. New X-ray structures: two parasite CRTs and a mutated version of HsCRT   

A dissection strategy was set up to restrict the molecular flexibility of the two parasite CRTs, since it prevents the initial crystallization step. As performed previously in the case of mammalian CRTs, this strategy involves partial or complete deletion of the flexible P domain and removal of the highly charged and flexible C-terminal extremity (Chouquet *et al.*, 2011 ▸; Kozlov *et al.*, 2010 ▸; Pocanschi *et al.*, 2011 ▸). Several trials were required to define a suitable TcCRT construct because simultaneously removing these flexible areas drastically reduced the molecular solubility and stability, as checked for example by fluorescent spectroscopy (not shown). SAXS studies also provided useful information about the residual flexibility that needs to be removed in order to generate protein constructs suitable for crystallization (not shown).

**Figure 1:**
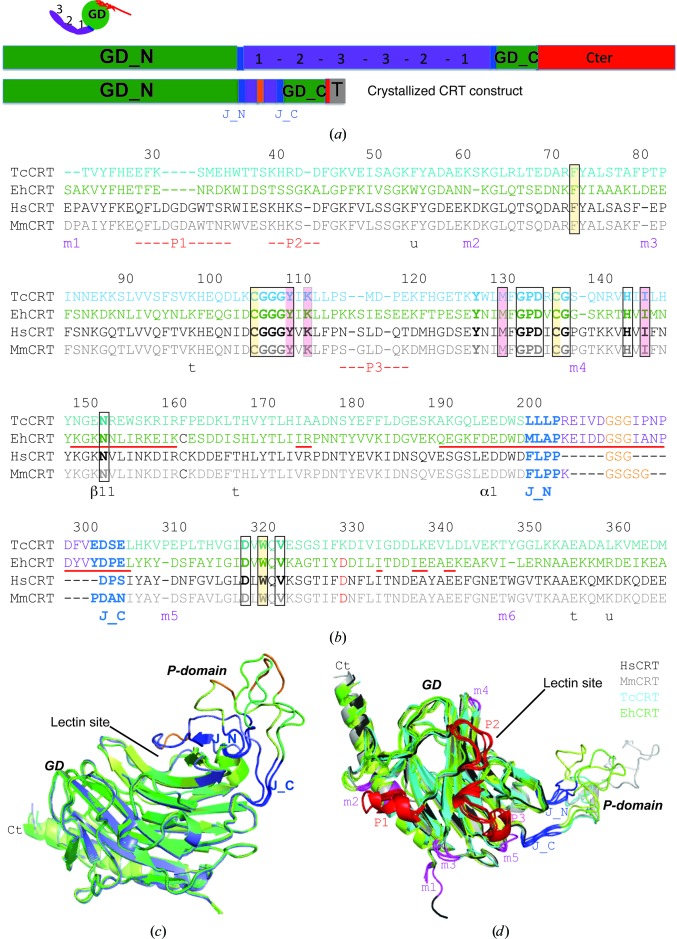
Sequences and structures of the recombinant CRT constructs. (*a*) CRT modular structure (top) and design of the crystallized construct (below). The GD domain (GD_N and GD_C, green) is fully conserved. In the P domain (magenta), 1 to 3 mark the hairpin sequence repeats. The truncation GSG linker is shown in orange, the small junction domain (J_N and J_C) linking the GD and P domains is in dark blue and the C-terminal His tag is labelled T (grey). (*b*) Structural alignment of mammalian (human, Hs; mouse, Mm) and parasite (Tc, Eh) CRTs. The identical residues of the ‘lectin site’ are boxed and coloured as in Fig. 3 ▸. The residues that are significantly reoriented or displaced in the closed-like EhCRT conformation (Fig. 2 ▸) are underlined (red) and the corresponding α1 and β11 secondary structures are indicated below the sequence alignment. The position of the main (P1 to P3, red) and the minor (m1 to m6, magenta) three-dimensional structure variations are indicated below, as well as the interactions (t, u) between the GD and the end of the C-­terminal α-helix (detailed in Supplementary Fig. S1). The aspartic calcium ligand, which is missing in TcCRT, is shown as a red D. TcCRT residue numbering is shown at the top. (*c*) The truncated P arm adopts various conformations in the superimposed EhCRT monomers. The ‘closed-like’ conformer is shown in blue, the ‘open’ conformers in various green/yellow colours and the linker is in orange. (*d*) Overall three-dimensional superposition of different CRTs. The same colour code is used as described above. Three different EhCRT open conformers are shown in shades of green. The corresponding PDB codes are 3rg0 (MmCRT; Pocanschi *et al.*, 2011 ▸) and 3pos (HsCRT; Chouquet *et al.*, 2011 ▸).

The first fragile TcCRT crystals were obtained by using a final construct comprising the intact globular and junction domains, together with a highly truncated P domain (Fig. 1 ▸ *a*). The junction domain connects the globular domain (GD) to the repeated motifs of the P domain (Pocanschi *et al.*, 2011 ▸). It comprises two stretches (J_N and J_C) of four consecutive residues (Figs. 1 ▸ *a* and 1 ▸ *b*, blue). To obtain this construct, the last 52 C-terminal residues of TcCRT were removed and 84 residues of the P-domain were replaced by a GSG linker (Figs. 1 ▸ *a* and 1 ▸ *b*). The diffraction quality of these crystals was slightly improved by adding a large amount (1 *M*) of glucose (Glc) to the crystallization droplet. A similar EhCRT construct was then produced and beautiful crystals were obtained. The X-ray structures of the corresponding TcCRT and EhCRT constructs were solved using molecular replacement and refined to 2.45 and 2.15 Å resolution, respectively (Table 1 ▸). For each molecular copy, the structure of the residual flexible P-domain segment had to be manually rebuilt into the corresponding electron-density map. More conformational variations of the P domain have been observed with the EhCRT construct, including a new ‘closed’ conformer (Fig. 1 ▸ *c*). As will be described later, this switch to a ‘closed’ conformation not only involves a new conformation of the truncated P domain, but also requires local conformational changes of the nearby junction domain and of one strand and one helix of the GD.

In addition, the structure of a mutated human CRT GD construct (HsCRT D71K) was solved and refined to 2.3 Å resolution (Table 1 ▸). The purpose of this HsCRT mutant was to disrupt an ionic bond observed in all previous crystal packing(s) that stabilizes CRT molecular arrays (Chouquet *et al.*, 2011 ▸). As expected from the mutation design, a new crystal form has been obtained, with ten independent copies now present in the asymmetric unit. The residues of the conserved lectin site (analysed below in §[Sec sec3.3]3.3) do not participate in the packing interactions in this new crystal form. Compared with the previous native HsCRT structure, the largest deviation was observed for its N-terminal extension, which, in the absence of interaction with a neighbouring lectin site, is more flexible and found in different orientations up to Val21. The part of the junction and linker region (LPGSGD) which was not initially defined in the wild-type (wt) model has been rebuilt in most of the copies. The final average root-mean-square deviation (r.m.s.d.) between the wt HsCRT structure and the different HsCRT D71K copies is very small (0.3 Å).

There is 40–45% sequence identity and about 60% similarity between the two parasite CRT constructs and their mammalian counterparts (Chouquet *et al.*, 2011 ▸; Kozlov *et al.*, 2010 ▸; Table 3 ▸). The r.m.s.d. is about between 1 and 1.5 Å, based on 240–250 aligned residues (Table 3 ▸). As will be detailed later on, the orientation of the junction domain appears to be conserved in the different species (Fig. 1 ▸ *d*). The conserved and stable CRT core comprises 223 residues. Species differences occur at ten sites in the GD (Fig. 1 ▸ *d*). The details of the three main P1 to P3 insertion/deletions, which are clustered on the same molecular face (Fig. 1 ▸), are shown in Supplementary Fig. S1. A major helical insertion in mammalian CRTs characterizes P1, whereas smaller EhCRT insertions occur in P2 and P3 (Fig. 1 ▸ *b*, Supplementary Fig. S1). P3 also features a one-residue deletion in TcCRT compared with mammalian CRTs. There is no free cysteine, as well as no calcium-binding site, in the TcCRT GD (Fig. 1 ▸ *b*).

### 3.2. The first example of a complex transition towards a ‘closed’ conformer   

**Figure 2:**
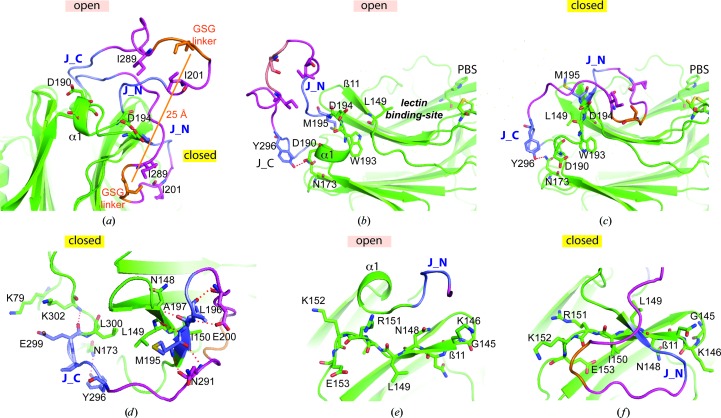
The closed-like EhCRT structure reveals a complex conformational rearrangement. (*a*) Superposition of the open and closed EhCRT conformers focusing on the different junction and P conformations. The same colour code is used as in Fig. 1 ▸(*a*). The linker serine residue moves 26 Å away. (*b*, *c*) Side-by-side comparison of the open and closed states, with emphasis on the tilt of the P domain. The stable Trp and disulfide bond at the edge of the lectin-binding site (PBS) are shown on the right. (*d*) Details of the junction interaction in the closed conformation. (*e*, *f*) Comparative side-by-side views of the unusual conformational rearrangements in α1, J_N and β11.

For the first time, a tilt of the truncated P domain onto the lectin site was observed in one EhCRT monomer, which thus appears as a ‘closed’ conformer (Figs. 2 ▸ *a* and 2 ▸ *c*). The transition to this new conformation appears to be quite complex and requires several concerted movements, as illustrated by a simple morphing decomposition in Supplementary Fig. S2 and Supplementary Movie S3. Large displacements are observed at the tip of the P domain, where the linker serine residue moves 26 Å away, with Ile201 and Ile289 also moving 18 and 22 Å away, respectively (Figs. 2 ▸ *a*, 2 ▸ *b* and 2 ▸ *c*). This conformational transition also involves additional concerted movements, such as unwinding of helix α1, remodelling of the junction domain and a twist in strand β11.

In this conformer, the orientation and interactions of the junction domain are thus significantly modified. The first J_N residue, Met195, is significantly displaced by 5.2 Å (Figs. 2 ▸ *b* and 2 ▸ *c*). This displacement is concerted with the unwinding of the preceding helix α1, with a 2 Å increase in the distance between Asp190 and Asp194 (Figs. 2 ▸ *a*, 2 ▸ *b* and 2 ▸ *c*). The space occupied by Met195 in the open conformer is now filled by leucines 149 and 300 in the closed conformer (Figs. 2 ▸ *b*, 2 ▸ *c* and 2 ▸ *d*). J_N forms a new short β-strand in the closed conformer, stabilized by hydrogen bonds between Met195 and Asn291 (P), Leu196 and Asn148 (GD), and Ala197 and Glu200 (P) (Fig. 2 ▸ *d*). Leu196 is also stabilized by hydrophobic contacts with Ile150 (Fig. 2 ▸ *d*). A kind of hinge motion occurs at Glu299, the last residue of J_C, which is anchored by interactions with the GD residues Lys79 and Lys302 (Figs. 2 ▸ *a* and 2 ▸ *d*). Although the position of Tyr296 (the first residue in J_C) is displaced by 4.5 Å, its hydrogen-bonding interaction with the GD Asn173 side chain is maintained (Figs. 2 ▸ *b*, 2 ▸ *c* and 2 ▸ *d*).

**Figure 3:**
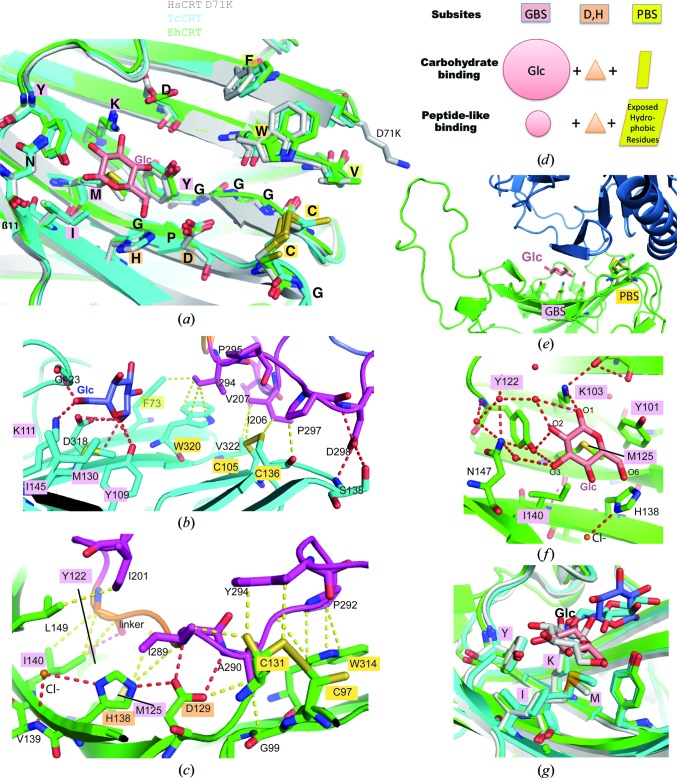
Dual substrate-binding properties of the conserved lectin domain. (*a*) Strong structural conservation of the lectin domain. The 20 identical calreticulin residues are shown as sticks. The glucose-binding (GBS) and peptide-like-binding (PBS) subsite residues are highlighted in pink and yellow, respectively. (*b*) First example of a dual substrate-binding interaction in TcCRT, with Glc–GBS (left) and peptide-like–PBS (right) interactions. (*c*) Details of lectin-domain interactions with the flexible P-domain of a neighbouring molecule in EhCRT. A Cl^−^ ion is shown in orange. (*d*) Simplified scheme illustrating the dual binding property of the lectin domain and how the GBS and PBS subsites can anchor interactions with glycans and peptides, respectively. (*e*) Global view of the large interface with the closed EhCRT conformer (dark blue), an exception among the lectin crystal-packing interactions. (*f*) Details of the interaction of Glc with GBS in EhCRT. (*g*) Superposition of Glc molecules bound in GBS in diverse contexts. The lectin-domain colour code is cyan for TcCRT, green for EhCRT and grey for HsCRT or MmCRT. The Glc colour code is salmon for EhCRT, grey for MmCRT (Kozlov *et al.*, 2010 ▸; PDB entry 3o0x) and light and dark blue for TcCRT. Several van der Waals and polar contacts are highlighted with yellow and red dashed lines, respectively.

To illustrate the local twist in strand β11 involved in the ‘open-to-closed’ conformational transition, major side-chain reorientations occurring after the Gly145 hinge are shown in Figs. 2 ▸(*e*) and 2(*f*). In the central part, the overall effect is quite similar to a one-residue shift in the β-strand, meaning for example that the main-chain carbonyl group of Leu149 moves to the position normally occupied by the corresponding group of Asn148 (Figs. 2 ▸ *e* and 2*f*). This is quite surprising because β11 is very close to the lectin site (Fig. 3 ▸ *a*), which, as detailed below, remains stable in all cases, with only a slight displacement of the two distal glycines 132 and 133 in the closed conformer (2.3 Å for Gly132).

### 3.3. Dual substrate-binding interaction properties in the conserved CRT lectin domain   

The concave lectin-binding surface is strikingly conserved in the CRT and CNX structures (Chouquet *et al.*, 2011 ▸; Kozlov *et al.*, 2010 ▸; Schrag *et al.*, 2001 ▸). Among the 20 residues that are strictly conserved in all CRTs, five glycines and one proline introduce a void in this concave face. The orientations of the other side chains are strictly maintained in all cases (Fig. 3 ▸ *a*), whether or not this domain is involved in crystal-packing interactions, and even in the closed-like EhCRT conformation. The disulfide bridge and the extended hydrogen-bonding network, which are conserved in all structures, are likely to play a crucial stabilizing role. A chloride ion coordinated by the side chain of His138 and the main chain of Val139 was observed in EhCRT (Figs. 3 ▸ *c* and 3 ▸ *f*) and TcCRT. Its identification has been reinforced by finding a clear anomalous peak at this position using EhCRT crystals and a wavelength of 2 Å (Supplementary Fig. S4).

As noticed in a previous study (Chouquet *et al.*, 2011 ▸), analysis of the lectin-site crystal-packing environment for the 21 different CRT molecules refined in this study reveals quite unusual interaction properties. There is no packing interaction on this surface in the mutated HsCRT crystal, and packing interactions in parasite CRT crystals only involve contacts with the flexible truncated P domains (which adopt different conformations in the different molecules and expose hydrophobic residues; Figs. 1 ▸ *c*, 3 ▸ *b* and 3 ▸ *c*), with the only exception of one interaction with the CRT in the closed-like conformation (Fig. 3 ▸ *e*). Thus, this part of the molecule is not prone to establishing ‘standard’ crystal contacts with native stable CRT surfaces. Two main subsites can be defined, according to their different kinds of interactions (Fig. 3 ▸ *d*, Supplementary Fig, S5): the glucose-binding site (GBS) and the peptide-like-binding site (PBS). The two subsites are not shaped as pockets but include hydrophobic residues, which might partly explain their enhanced interaction propensities. The choice of the abbreviations GBS and PBS will be addressed as part of the discussion. The GBS is mainly delineated by the conserved Met, Ile, Lys and Tyr residues (Fig. 3 ▸, Supplementary Fig. S5), whereas the PBS includes the disulfide bridge and the tryptophan at the edge of the lectin site (common to CRTs and CNXs), as well as Phe or Val, with the latter being conserved only in CRTs (Fig. 3 ▸, Supplementary Fig. S5).

Very intriguingly, a very reproducible electron density was observed within the GBS in the first EhCRT crystal form that was not related to the crystallization medium. This density has been interpreted as a Glc molecule sandwiched between monomers *A* (lectin site) and *C* (closed conformer) (Figs. 3 ▸ *e* and 3 ▸ *f*, Supplementary Fig. S6). This Glc molecule lies flat in the shallow cavity above Met and Ile, and is further stabilized by water-mediated hydrogen bonds. Three Glc hydroxyl groups are directly hydrogen-bonded to the Lys103 (O1) and Tyr122 (O2 and O3) side chains (Fig. 3 ▸ *f*). This is highly similar, despite a reverse orientation, to the position of the Glc moiety previously observed in the interaction between MmCRT and the Glc_1_Man_3_ tetrasaccharide (Fig. 3 ▸ *g*, Supplementary Fig. S5; Kozlov *et al.*, 2010 ▸). This is in contrast to the poorly stabilized binding of the Glc molecule in the context of the TcCRT hybrid interactions observed when a large amount of Glc was present in the crystallization medium (Fig. 3 ▸ *b*, Supplementary Fig. S7). The Glc positional variations in this case reflect the poor millimolar affinity of CRT for free Glc, as well as competitive acetate binding and steric restrictions from the neighbouring PBS interaction (Fig. 3 ▸ *b*). In this configuration, the O1, O5 and O6 Glc hydroxyl groups are stabilized by the side chains of Tyr109, Lys111, Gly123 and Asp318, and the Glc molecule binds more distantly from the conserved Ile and Met residues of the lectin site (see Fig. 3 ▸ *g*). As will be discussed, GBS can also interact with amino acids in the absence of Glc, as seen for example with the linker in Fig. 3 ▸(*c*) or Supplementary Fig. S5.

Three representative examples of PBS interactions will be described. As a common hallmark, they show numerous hydrophobic contacts. The first example comes from the TcCRT study, where the PBS directly interacts with the following hydrophobic residues of the truncated P arm: Ile206, Val207, Ile294 and Pro297 (Fig. 3 ▸ *b*). The following example, from the EhCRT study, shows a larger interaction surface (Fig. 3 ▸ *c*). In this case, the PBS stabilizes the hydrophobic side chains of Ile289, Ala290, Pro292 and Tyr294, whereas the GBS interacts with Ile289 and the linker peptide. In between, Asp129 stabilizes the main-chain N atoms of Ile289 and Ala290 through two hydrogen bonds. The last example comes from a very special case in which a Glc molecule is sandwiched between the two GDs (Fig. 3 ▸ *c*), and thus might represent an exception (Supplementary Fig. S8). Nevertheless, direct PBS van der Waals contacts also occur in this case between Cys131 and Ile168/Arg170, as well as between Trp314 and Pro171/Asn82. This interaction involves addional polar and electrostatic interactions with the lectin domain, as well as a contribution from the associated truncated P domain. Interestingly, among the residues of the closed conformer which interact with the lectin site, several adopt a different position/conformation compared with the open conformer (highlighted by a blue label in Supplementary Fig. S8). This suggests that some kind of induced fit could be required before binding to the lectin site, which in turn finally binds to an altered conformation (here of CRT).

### 3.4. Insights into the overall flexibility of the open conformer   

SAXS was used to study the overall shape and flexibility of several TcCRT constructs. SAXS experimental data sets were recorded at four concentrations for both the full-length TcCRT molecule and the crystallized TcCRT construct (Table 2 ▸).

The data sets collected from the crystallized TcCRT construct did not show significant changes (<3%) in the *R*_g_ and *I*(0) throughout the dilution series (Table 2 ▸). Consequently, we merged the data collected at 1.8 mg ml^−1^ (range 1–605) and 11.15 mg ml^−1^ (605–1036) to determine the overall structural parameters (Supplementary Fig. S9). The Guinier analysis led to an *R*_g_ value of 2.17 nm with a good linear fit (fidelity of 0.99) in the low-*s* region (*sR*_g_ < 1.3; Table 2 ▸, Supplementary Fig. S9). A similar real-space *R*_g_ value of 2.12 nm was obtained with *GNOM* (Table 2 ▸). The pair distance distribution function *P*(*r*) exhibited a perfect Gaussian shape, in agreement with the fact that the construct became mainly globular after truncation of the P domain (Fig. 1 ▸ *d*, Supplementary Fig. S9*c*). The convergence of the Kratky plot back to the baseline also suggests that the crystallized construct does not contain any major flexible domains (Supplementary Fig. S9*d*).

**Figure 4:**
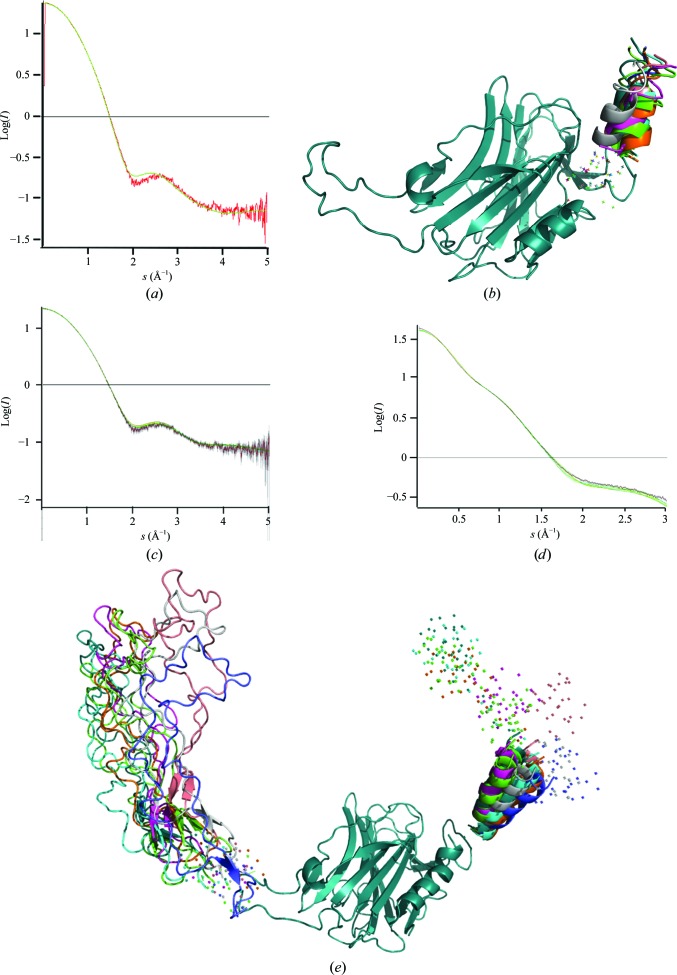
Solution structure of TcCRT: full-length molecule and crystallized construct. (*a*) The comparison between the theoretical (*CRYSOL*) and experimental scattering curves of the crystallized TcCRT construct shows a difference in the Porod domain, which suggests that the relative orientation of the domains is different in the solution structure compared with the X-ray structure. (*b*) Different orientations of the C-­terminal helix proposed by *BUNCH* improved the fit (*c*) in this case. The ten best models are shown here (with χ_2_ values between 0.96 and 1.03). (*d*, *e*) In the case of the full-length TcCRT solution structure, combinations of C-terminal and P-domain reorientations with *BUNCH* improve the curve fit to the Porod domain (0.3 nm). The ten best results are shown here (with χ_2_ values between 3.95 and 5.34). Local predictions for undefined segments in the initial protein models are shown by dots in (*b*) and (*d*).

Slight differences in the medium-*q* region appear when comparing the experimental curve and the theoretical solution scattering curve calculated from the TcCRT X-ray structure using *CRYSOL* (Petoukhov *et al.*, 2012 ▸), which suggests variations of the relative orientations of two subdomains in the solution structure (Fig. 4 ▸ *a*). Several hinge locations (the C-terminal helix, the junction of the P domain) were tested using the *BUNCH* software (Petoukhov *et al.*, 2012 ▸), and only the hinge corresponding to the C-terminal fluctuations significantly improved the fit to the experimental data (Figs. 4 ▸ *b* and 4 ▸ *c*). However, there was no convergence towards a unique solution, which is consistent with the hypothesis that the C-terminal extremity of the helix oscillates freely. Fig. 4 ▸(*c*) illustrates the variation of the C-terminal orientations in several *BUNCH* models that fit the experimental curve better than the original X-ray model. Interestingly, subtle C-terminal positional fluctuations are also consistently observed in the CRT X-ray structures (Supplementary Fig. S1*d*). They correlate with the decrease in stabilizing inter­actions between the C-terminal helix and the GD. Although the level of sequence conservation in the C-terminal part is far lower than in the P and lectin domains, the last two conserved sets of interactions between the C-terminal helix and the GD can be delineated (noted u and t in Fig. 1 ▸ *b* and Supplementary Fig. S1*d*).

Comparison of the *R*_g_ and *I*(0) values for each data set for the full-length protein showed a decrease of 20% at the two highest concentrations (Table 2 ▸), suggesting that repulsive inter­actions begin to occur at high concentrations. Therefore, we merged the Guinier region of the data collected at 1.63 mg ml^−1^ (range 1–105), the Porod region of the data collected at 7.05 mg ml^−1^ (range 106–335) and the high-*q* region of the data collected at 10.4 mg ml^−1^ (range 336–1036) to compute the overall structural parameters (Supplementary Fig. S10*a*, Table 2 ▸). The Guinier analysis showed a good linear fit (fidelity of 0.99) in the low-*s* region (*sR*_g_ < 1.3) and suggested an *R*_g_ of 3.74 nm (Table 2 ▸, Supplementary Fig. S10*b*). Besides, the real-space *R*_g_ and the longest interatomic distance (*D*_max_) calculated by the indirect Fourier transformation with *GNOM* were 3.86 and 13.09 nm, respectively (Table 2 ▸). The pair distance distribution function *P*(*r*) shows a Gaussian shape followed by a shoulder in the longer distance region, which suggests the presence of both a globular domain and an elongated domain, in agreement with the expected structure (Fig. 4 ▸ *d*, Supplementary Fig. S10*c*). The parabolic shape including a shoulder of the Kratky plot confirms a molecular shape comprising two domains, while the divergence from the baseline at higher *s* values confirms the presence of a flexible domain (Supplementary Fig. S10*d*).

To investigate the solution structure of full-length TcCRT, the approach described above was extended to a hybrid model including the X-ray structure as well as the modelled P arm and C-terminal end. Several hinge positions in the P and C domains were tested in this case. As illustrated in Figs. 4 ▸(*d*) and 4 ▸(*e*), the best fits were obtained using the C-terminal hinge (determined for the crystallized construct), together with that following the junction domain, leading to a set of models in agreement with the experimental data up to 0.3 nm^−1^. The results suggest that the P arm is swaying apart from the junction domain (Fig. 4 ▸ *d*). Moreover, the P domain seems to remain in an open conformation in the TcCRT solution structure (Fig. 4 ▸ *d*).

### 3.5. A rigid junction between the lectin and P domains (open form)   

**Figure 5:**
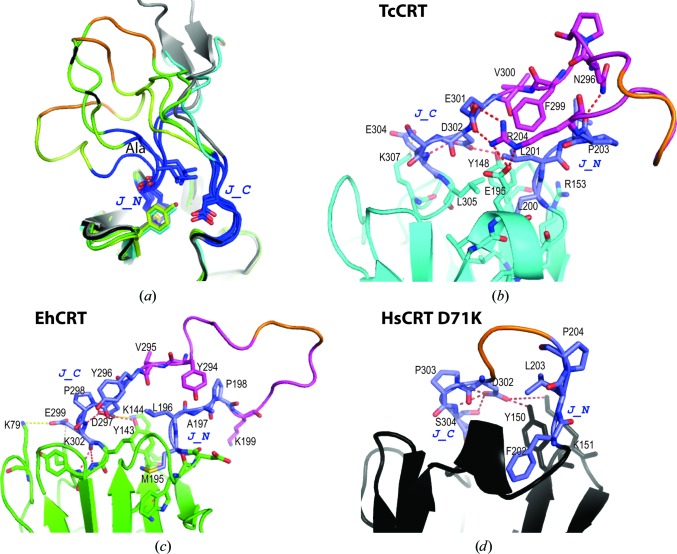
Conserved junction structure, interactions and orientation (‘open’ form). (*a*) Global view of the junction superposition, highlighting its main conserved structural features. Ala shows the mean position of the unusual alanine in EhCRT. (*b*) Junction interaction details in TcCRT. (*c*) Junction interaction details in EhCRT, open conformation. (*d*) Junction interaction details in HsCRT D71K. Colour code: the junction is shown in dark blue, the truncated P domain in magenta, the linker in orange, TcCRT in cyan, EhCRT in green, HsCRT D71K in black and MmCRT (PDB entry 3rg0) in grey in (*a*).

The TcCRT SAXS analysis above suggests that the junction domain is stable in the solution structures. This domain appears to also be stable in the different X-ray structures (TcCRT, MmCRT and HsCRT D71K mutant), which allows us to decipher common junction features (Fig. 5 ▸). This conserved set of interactions between the junction and the GD stabilizes a similar orientation of the P domain in all CRTs (Fig. 5 ▸ *a*), which differs from that observed in the CNX structure (Schrag *et al.*, 2001 ▸; Pocanschi *et al.*, 2011 ▸).

The N-terminal sequence of the junction (J_N) is highly hydrophobic (Figs. 1 ▸ *b* and 5 ▸). The first residue (Leu200/Met195/Phe202) is anchored into the GD, holding the junction in a perpendicular orientation (Figs. 5 ▸ *b*, 5 ▸ *c* and 5 ▸ *d*). The following leucine residue (201, 196 and 203) contributes to the junction core. The last two J_N residues are often prolines, except in EhCRT and TcCRT, where only the last proline is conserved (Fig. 1 ▸ *b*). The first proline residue is replaced by an alanine in EhCRT (indicated Ala in Fig. 5 ▸ *a*), which introduces local flexibility, and the bending introduced by the following proline residue further amplifies this hinge movement.

The C-terminal part of the junction (J_C) starts with a nonconserved (Glu/Tyr/Pro) residue. The following conserved aspartate residue (302, 297 and 302 in TcCRT, EhCRT and HsCRT, respectively) always interacts with the hydroxyl group of a conserved tyrosine side chain of the GD (Fig. 5 ▸). This aspartate residue also interacts with a basic side chain in the P domain (Arg204 in Tc; Fig. 5 ▸ *b*) or the GD domain: Lys144 in EhCRT (Fig. 5 ▸ *c*), Lys151 in MmCRT or HsCRT (Fig. 5 ▸ *d*). In the case of TcCRT and EhCRT, the last J_C acidic residue interacts with a basic residue of the GD: Lys307 (Fig. 5 ▸ *b*) and Lys79 (Fig. 5 ▸ *c*), respectively.

## 4. Discussion   

The present combination of X-ray and SAXS analyses has provided consistent insights into the flexibility of the open form of CRT. This flexibility mainly involves swinging of the P domain after the rigid junction, as well as oscillations of the free C-terminal end (Fig. 4 ▸ *d*). This molecular flexibility, which is known to be a limiting step in the X-ray structural studies of CRTs, has been successfully reduced in the two parasite CRTs by generating a recombinant construct suitable for X-ray studies and including a truncated P domain (Fig. 1 ▸). Since the same design has been used to study the two parasite structures, it might also be useful to solve other CRT subgroups, such as plant CRTs, plant parasite CRTs or the testis-specific mammalian CRT2 isoform (Persson *et al.*, 2002 ▸). This would help to better define what it is that drives the functional differences of CRTs, an area which is still expanding (Johnson *et al.*, 2001 ▸; Qiu *et al.*, 2012 ▸; de Bruyn *et al.*, 2015 ▸; Xiang *et al.*, 2015 ▸) and possibly also to provide structural insights into some CRT2-mutation related diseases (Chiu *et al.*, 2007 ▸). Coming back to the two parasite CRTs studied here, if one needs to block them without affecting the host CRT, selective inhibitory peptides or antibodies need to be developed, which is only possible if one can identify distinctive structural features in the parasite CRTs. Such distinctive features cluster on one face of the molecule (shown in red in Fig. 1 ▸ *d* and detailed in Supplementary Fig. S1), suggesting a functional evolutionary pressure and providing a potential selective target for future therapeutic strategies. These species differences could also help to better understand the high anti-angiogenic potential of TcCRT and how it favours wound healing (Ramírez-Toloza *et al.*, 2015 ▸; Ignacio Arias *et al.*, 2015 ▸).

The comparison of phylogenetically distant species is also helpful to obtain further insights into the most conserved structural features of CRTs, such as the junction domain or the lectin site (Figs. 1 ▸ *d*, 3 ▸ *a* and 5 ▸ *a*). For the first time, the concave lectin surface is shown to simultaneously bind a Glc molecule and a protein component (Figs. 3 ▸ *b* and 3 ▸ *e*), which provides structural support to better understand its dual substrate-binding properties (Wijeyesakere *et al.*, 2013 ▸; Hirano *et al.*, 2015 ▸). In line with our previous observations (Chouquet *et al.*, 2011 ▸), this leads us to define two main GBS and PBS subsites, which would provide anchoring interactions with various ligands (Fig. 3 ▸ *d*). The choice of the abbreviation GBS (for glucose-binding subsite) is intended to reflect the fact that until now Glc has only been seen to interact with this subsite (Fig. 3 ▸ *g*) and to remind that interaction of a glycosylated component with CRT absolutely requires a Glc moiety, because binding is lost as soon as Glc is removed (see, for example, Amin *et al.*, 2011 ▸). This latter property drives the glucose-trimming and glucose-tagging process that is known to be used in the CNX/CRT cycle. Although GBS residues are already known to interact with Glc, this study suggests possible versatility with respect to their fine interaction details (Fig. 3 ▸ *g*). The choice of the abbreviation PBS (for peptide-like-binding subsite) is intended to reflect its van der Waals interaction propensity towards exposed hydrophobic residues, as seen in several independent crystal-packing interactions with the flexible truncated P domain in this study (Figs. 3 ▸ *b* and 3 ▸ *c*). These observations are fully consistent with the recent experimental evidence of a polypeptide-binding site in the vicinity of the conserved lectin-site histidine residue (in Fig. 3 ▸ *a*) using a fluorescent probe fastened at this position (Wijeyesakere *et al.*, 2013 ▸). However, several subsites need to be engaged to achieve significant affinity, since they are not shaped as deep binding pockets (Figs. 3 ▸ *a* and 3 ▸ *d*). For example, the affinity of CRT towards free Glc is know to be very low (in the millimolar range) and the affinity of CRT jumps from disaccharides to trisaccharides (×45) and from trisaccharides to tetrasaccharides (×2) (Kapoor *et al.*, 2003 ▸). This requirement for binding by simultaneous subsites translates in the context of crystal-packing observations into a possible switch in the observed binding specificity, featuring a kind of synergy between the two proximal subsites by bringing an additive contribution to the other subsite (Fig. 3 ▸ *d*). For example, the GBS can bind amino acids in the absence of Glc as an additive contribution to PBS binding (Fig. 3 ▸ *c* and Supplementary Fig. S5). Conversely, the PBS has been described to interact with a mannose moiety in the context of the ‘classical’ lectin site interaction with the long model substrate G1M3 (Glc-Man_3_; Kozlov *et al.*, 2010 ▸; Supplementary Fig. S5). Such a possible switch in subsite specificity introduces a layer of complexity when trying to decipher such versatile and dual substrate-binding properties. This probably contributed to and explains the controversy about the localization of a putative chaperone peptide-binding site (Chouquet *et al.*, 2011 ▸; Pocanschi *et al.*, 2011 ▸; Wijeyesakere *et al.*, 2013 ▸). The proposed interaction scheme (Fig. 3 ▸ *d*), with a specificity driven by the main GBS or PBS anchoring interactions, differs from the commonly accepted schemes because the glycan-binding site overlaps with a putative peptide-binding site. However, this scheme is the only one which can reflect the following properties of CRT. (i) CRT not only recognizes the carbohydrate moiety, but also the proximal aglycone ligand part (Hirano *et al.*, 2015 ▸); this has been assessed by observing increased CRT binding correlated to increased aglycone hydrophobicity when comparing several G1M9-derived ligands. (ii) The synthetic glycan G1M3 can inhibit glycan-dependent and glycan-independent calreticulin–substrate interactions (Wijeyesakere *et al.*, 2013 ▸). (iii) Glycan-dependent and glycan-independent substrate inter­actions can be sensed by a fluorescent probe introduced at the position of the conserved His (Wijeyesakere *et al.*, 2013 ▸). The proposed scheme and the main subsites identified here also perfectly fit with the known effects of several point mutations (corresponding to GBS or PBS residues) onto the chaperone function in the CRT/CNX family (Groenendyk *et al.*, 2011 ▸; Martin *et al.*, 2006 ▸; Liu & Li, 2013 ▸). These subsites indeed comprise residues that are conserved in all CRTs and CNX sequences, with the conservation between the CRTs and CNX members being stronger for the GBS.

Both the X-ray and SAXS studies tend to show that the junction domain remains rigid and stable in the CRT open form. Fortunately, however, a complex conformational transition towards CRT closure could be observed for the first time (Figs. 1 ▸ *c* and 2 ▸ *a*). It features a complete reorientation and restructuration of the J_N junction segment, combined with other unusual structural rearrangements (Fig. 2 ▸). Experimental evidence supporting the existence of such ‘open’ and ‘closed’ conformations has recently been provided using two engineered fluorescent probes: one in the vicinity of the lectin site and one at the tip of the P domain (Wijeyesakere *et al.*, 2013 ▸). Although the context is different in our study, a conformational transition similar to that observed in EhCRT would be fully consistent for two main reasons. Firstly, there is high sequence conservation between EhCRT and mammalian CRTs in the area involved in the conformational rearrangement, especially the junction, the β11 strand and the α1 helix (Figs. 1 ▸ *b* and 2 ▸). Secondly, this hypothesis would support the fact that glycosylated substrates favour the open conformation in MmCRT (Wijeyesakere *et al.*, 2013 ▸): Glc could indeed restrict the conformational change towards the closed conformer, especially the twist in β11 illustrated in Figs. 2 ▸(*e*) and 2 ▸(*f*), because the conserved Asn in β11 is engaged in stabilization of the Glc molecule (Fig. 3 ▸ *f*; Kozlov *et al.*, 2010 ▸). On another note, the fact that helix α1 is altered in the closed conformer is quite interesting considering that (i) α1 corresponds to the proposed binding site of HsCRT for the gamma-aminobutyric acid receptor-associated protein (GABARAP; Thielmann *et al.*, 2009 ▸) but (ii) a conformational transition seems to be required to expose its central Trp residue, a major ligand of GABARAP, which is buried in the native structure.

Finally, finding a Glc molecule sandwiched between the lectin site of EhCRT and the closed conformer (Fig. 3 ▸ *e*, Supplementary Figs. S6 and S8) was completely unexpected because Glc was never added during the experiment. This suggests that Glc was partly bound to EhCRT during all steps of the purification process. It could thus be interesting to possibly check in the future whether CRT could carry Glc in a similar sandwich configuration under physiological particular conditions in which the concentrations of both Glc and CRT are increased, such as diabetes (Boden *et al.*, 2011 ▸; Sage *et al.*, 2012 ▸).

In conclusion, this structural study leads to a new hypothesis about possible CRT conformational rearangements, as well as a new interaction scheme supporting the controversial dual binding properties of CRT lectin sites (Fig. 3 ▸ *d*). This complements recent experimental evidence of such hybrid binding specificity and flexibility obtained by others using fluorescence and competition assays (Wijeyesakere *et al.*, 2013 ▸). Furthermore, this study also provides wider perspectives in the field of CRT structure–function relationships as well as in future therapeutic investigations of parasite CRTs.

## Supplementary Material

PDB reference: *Entamoeba histolytica* calreticulin, 5hca

PDB reference: 5hcb

PDB reference: *Trypanosoma cruzi* calreticulin, 5hcf

PDB reference: human calreticulin, D71K mutant, 5lk5

Supplementary Figures S1, S2 and S4-S10.. DOI: 10.1107/S2052252516012847/dc5006sup1.pdf

Supplementary Movie S3: Simple morphing illustrating the conformational changes required to go from the open to the closed conformer of EhCRT. Some side chains are shown: the Trp central to helix 1 and several side-chains in strand b11. DOI: 10.1107/S2052252516012847/dc5006sup2.gif

## Figures and Tables

**Table 1:** CRT data collection Values in parentheses are for the outermost shell.

	TcCRT	EhCRT, form 1	EhCRT, form 2	HsCRT D71K
Data collection				
ESRF beamline	ID29	ID23-EH1	ID23-EH2	ID23-EH1
Wavelength (Å)	0.97625	0.97625	0.87260	0.9763
Space group	*P*1	*P*2_1_2_1_2_1_	*P*4_2_2_1_2	*P*4_2_
Unit-cell parameters				
*a* (Å)	79.3	74.4	149.3	197.0
*b* (Å)	79.4	143.4	149.3	197.0
*c* (Å)	85.1	171.6	117.0	67.8
α (°)	95.6	90	90	90
β (°)	98.7	90	90	90
γ (°)	119.9	90	90	90
Resolution range (Å)	82–2.45 (2.60–2.45)	43–2.15 (2.20–2.15)	47.2–2.90 (3.07–2.90)	100–2.28 (2.36–2.28)
Observed reflections	126356 (19089)	354918 (22073)	263346 (42163)	480759 (20853)
Unique reflections	58978 (9064)	96632 (6384)	29847 (4641)	114120 (8478)
Multiplicity	2.14 (2.1)	16.07 (3.48)	8.82 (9.08)	4.04 (1.8)
Completeness (%)	92 (86.6)	99.2 (99.1)	99.5 (97.8)	95.8 (73.1)
〈*I*/σ(*I*)〉	7.14 (1.58)	13.40 (1.57)	16.08 (1.82)	9.41 (2.77)
*R*_merge_ (%)	8.3 (52.2)	5.9 (88.5)	10.3 (90.6)	11.8 (49.7)
CC_1/2_	99.3 (60.6)	99.8 (53.6)	99.8 (70.1)	99.5 (84.4)
Wilson *B* factor (Å^2^)	49	51.5	72.4	25.9
Refinement				
Resolution range (Å)	47.7–2.45	43–2.15	47.2–2.9	47–2.3
*R*_work_/*R*_free_ † (%)	22.29/25.66	19.49/22.23	19.01/21.73	21.02/24.68
R.m.s.d., bond lengths (Å)	0.004	0.007	0.012	0.016
R.m.s.d., angles (°)	0.837	0.824	1.516	1.68
Mean *B* factor (Å^2^)	48	52	88	24
PDB code	5hcf	5hca	5hcb	5lk5

† 5% of the structure factors were isolated to monitor *R*_free_.

**Table 2:** SAXS analysis of full-length TcCRT and its crystallized construct: parameters derived from the data collected at different concentrations (in mg ml^−1^) and from the merged scattering curve

	Full-length TcCRT					Crystallized TcCRT construct				
Curve	Merged	1.63	3.09	7.05	10.40	Merged	1.80	4.73	5.83	11.15
*I*(0)†	42.8 ± 0.05	42.9 ± 0.07	42.8 ± 0.04	38.7 ± 0.02	34.5 ± 0.03	24.0 ± 0.01	24.8 ± 0.03	24.5 ± 0.01	24.6 ± 0.01	24.79 ± 0.01
Linear segment†	12–44	24–61	11–54	12–65	12–47	22–119	28–101	20–173	22–132	16–93
*R*_g_ † (nm)	3.74 ± 0.01	3.79 ± 0.01	3.73 ± 0.01	3.37 ± 0.03	3.10 ± 0.01	2.17 ± 0.01	2.17 ± 0.01	2.11 ± 0.001	2.10 ± 0.001	1.98 ± 0.06
*R*_g_ ‡ (nm)	3.86	3.92	3.82	3.58	2.88	2.12	2.18	2.12	2.11	2.05
*D*_max_ ‡ (nm)	13.09	13.11	12.78	11.55	17.96	7.38	7.43	7.06	7.34	6.62
Porod volume‡ (nm^3^)	63.91	90.43	65.72	77.29	74.44	52.64	57.19	55.44	51.45	48.23

† Guinier analysis using *PRIMUS*.

‡ Indirect Fourier transform analysis using *GNOM*.

**Table 3:** CRT structure-comparison statistics The corresponding PDB codes are 3o0w for MmCRT and 3pos for HsCRT.

	No. of aligned residues	R.m.s.d. (Å)	Sequence identity (%)	Sequence similarity (%)
EhCRT/TcCRT	250	1.5	41	61
TcCRT/MmCRT	240	0.9	40	58
EhCRT/MmCRT	241	1.1	45	62
HsCRT/MmCRT	248	0.3	93	98
