# Supplementary material for: Structures of parasite calreticulins provide insights into their flexibility and dual carbohydrate/peptide-binding properties
Source: IUCrJ. 2016 Sep 14;3(Pt 6):408–19. doi: 10.1107/S2052252516012847 (PMC5094443; doi:10.1107/S2052252516012847)
Supplement: Supplementary file 1 [file m-03-00408-sup1.pdf]

IUCrJ

Volume 3 (2016)

Supporting information for article:

Structures of parasite calreticulins provide insights into their flexibility and dual carbohydrate/peptide binding properties

Christophe Moreau, Gianluca Cioci, Marina Iannello, Emmanuelle Laffly, Anne Chouquet, Arturo Ferreira, Nicole M. Thielens and Christine Gaboriaud

Fig. S1, related to Fig. 1

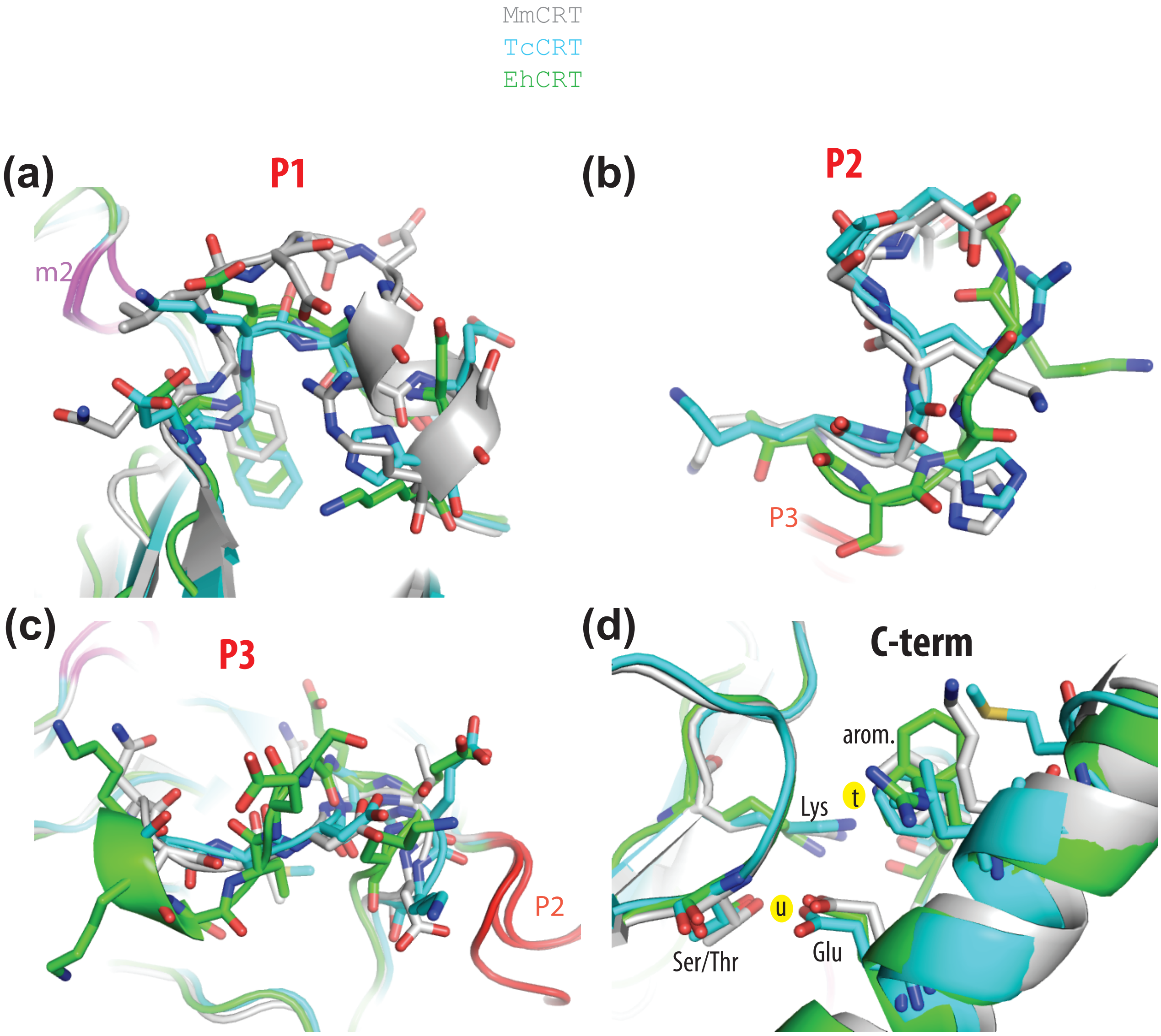

(a)-(c): Details of the major insertion/deletions shown in Fig. 1b and 1d.
(d): Details of the last conserved GD/helix interactions stabilizing the proximal part of the C-terminal helix (noted t and u in Fig. 1b).

Fig. S2, related to Figure 2 and to the animated gif S3

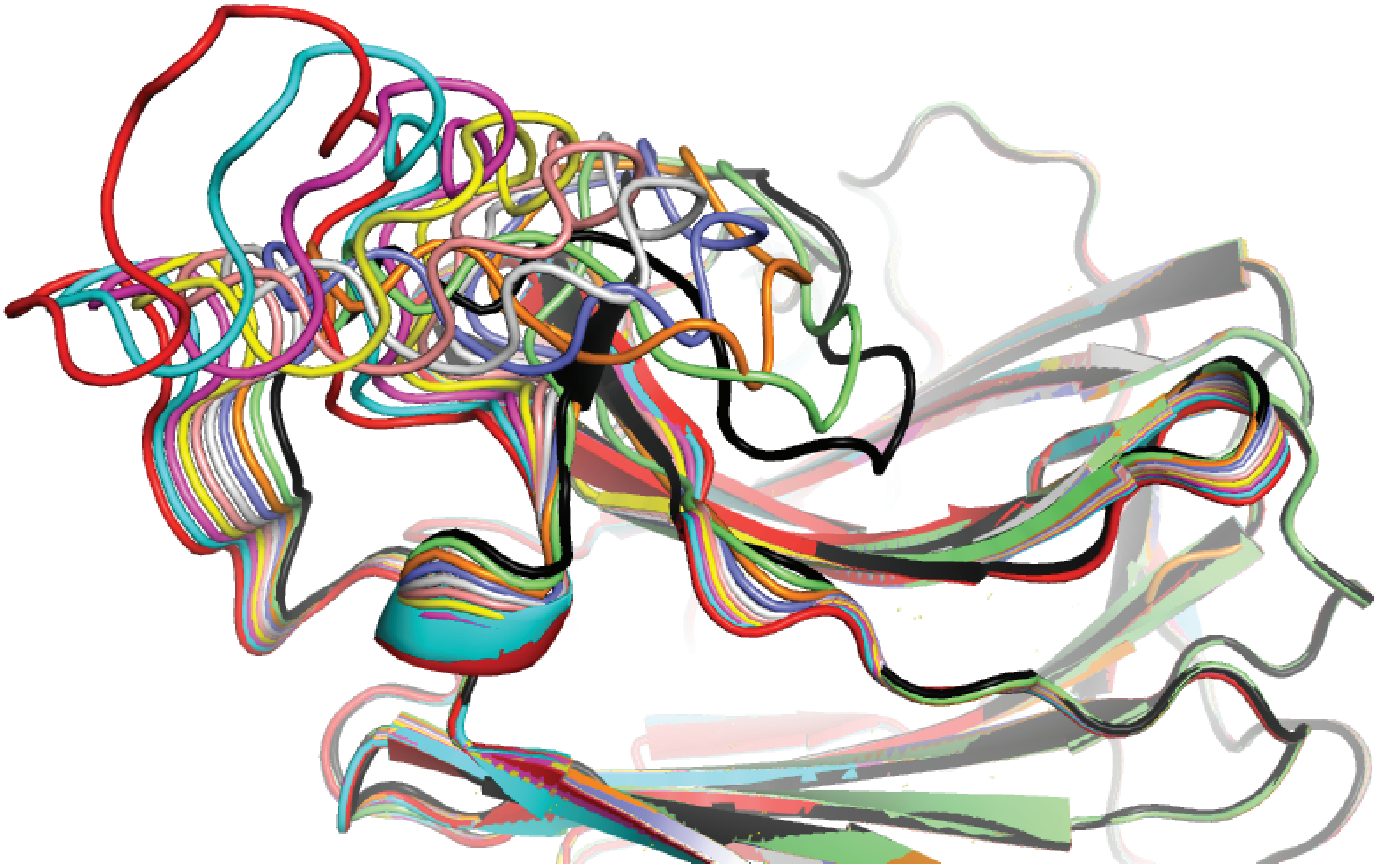

Simple morphing decomposition of the transition between the open (red) and the closed (black) conformers of EhCRT.

Fig. S4, related to Figure 3

(a)

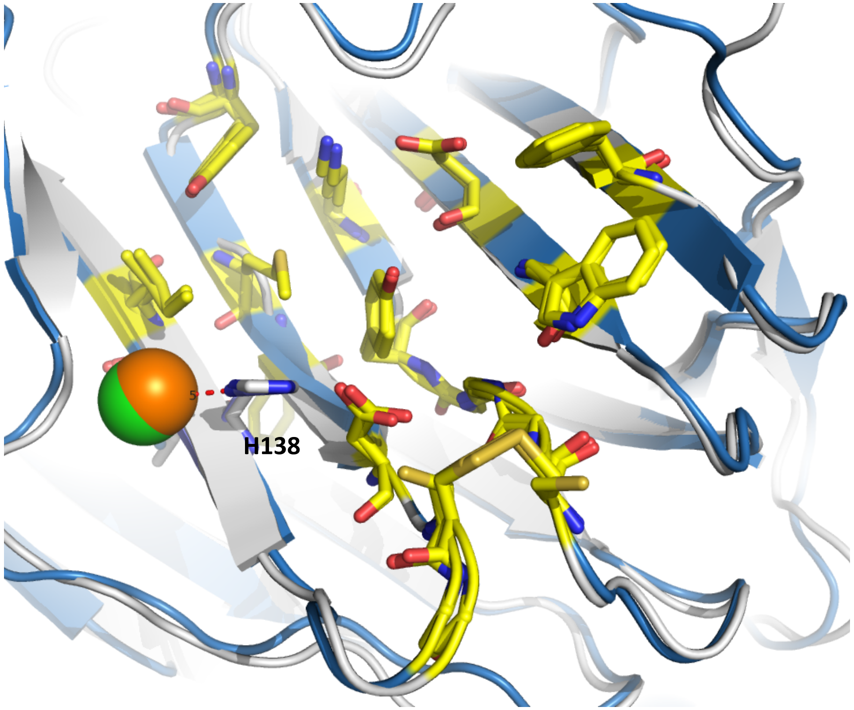

A Cl– ion has been detected in the vicinity of the lectin site of EhCRT (green) and TcCRT (orange).

(b)

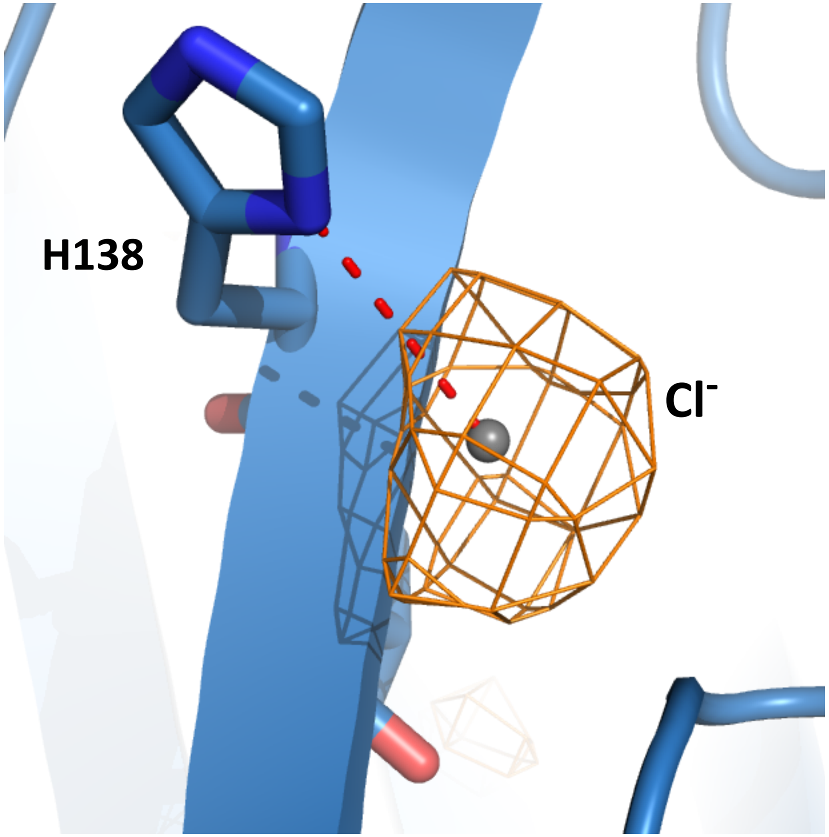

Anomalous difference map contoured at a 5σ level. A wavelength of 2 Å was used to collect the dataset on EhCRT crystals.

Fig. S5, related to Figure3

(a) GBS

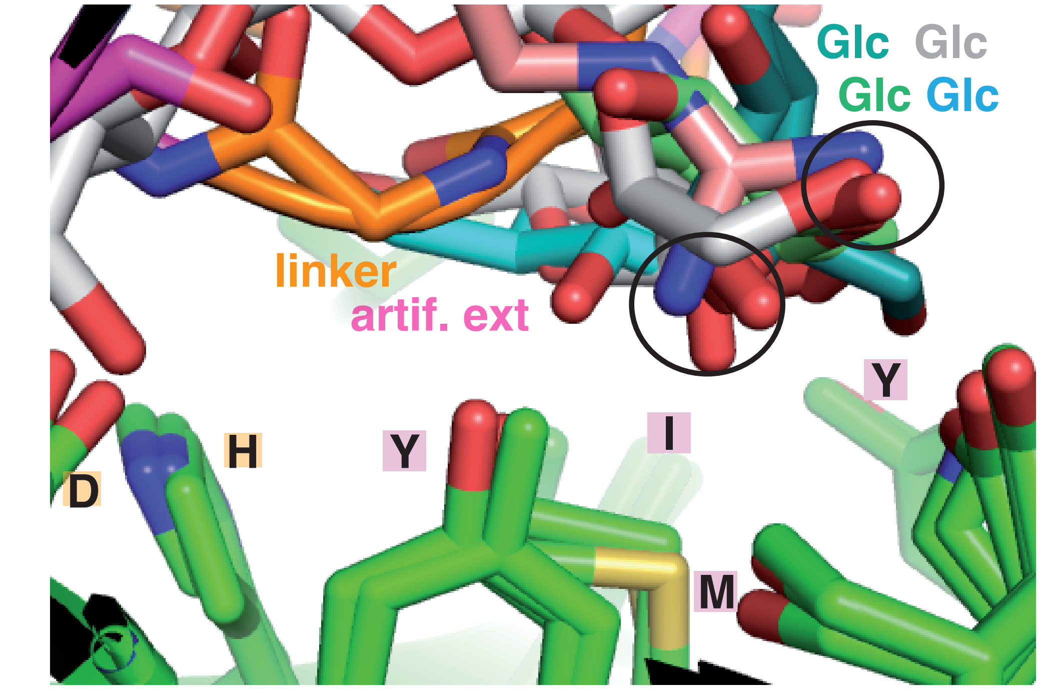

(b) PBS

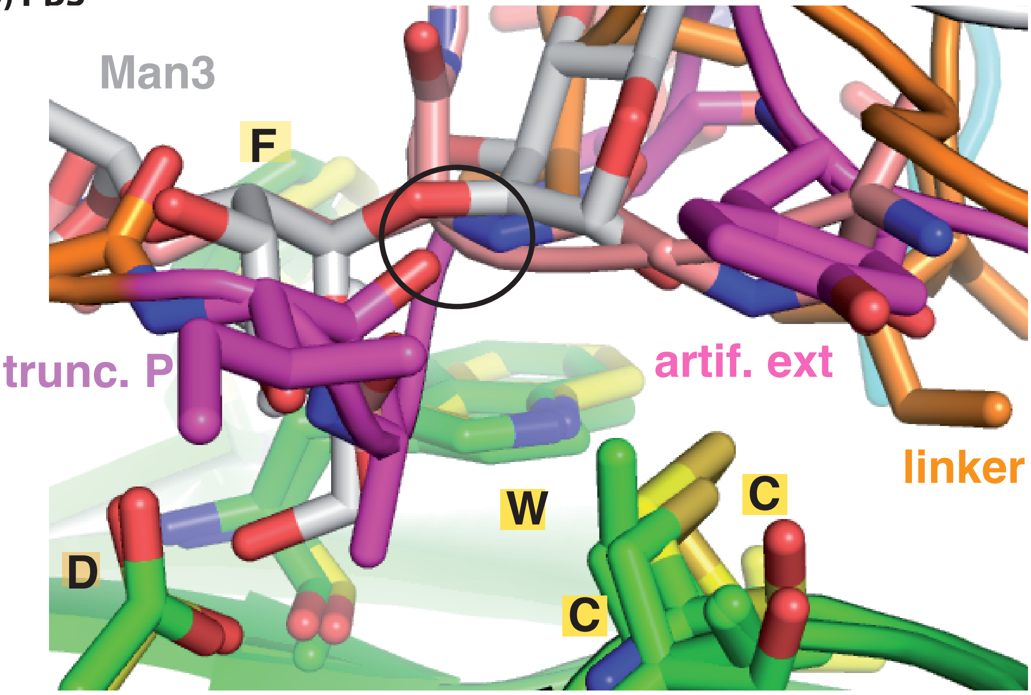

Selected superposition details of several GBS and PBS interactions.
Circles highlight some interactions shared by carbohydrates or peptides
(a): Glc in GM3/MmCRT (3O0W), Glc & Glc (TcCRT), Glc (EhCRT), linker (EhCRT), artificial extension from HsCRT (salmon color, 3POW).
(b): Man3 in GM3/MmCRT (3O0W), artificial extension from HsCRT (salmon color, 3POW), linker (TcCRT (right), EhCRT (left)), truncated P (EhCRT)

Fig. S6, related to Figure 3

(a)

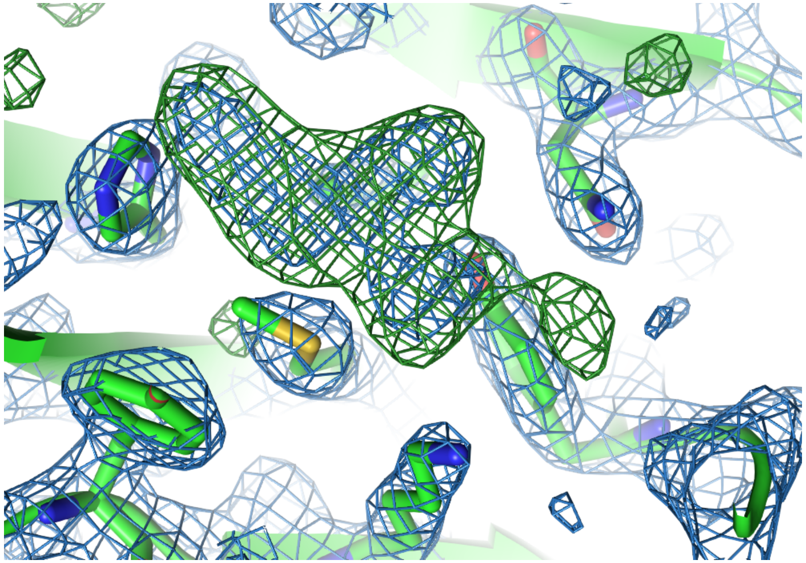

An additional electron density between the two EhCRT GD domains in the Fo-Fc map (green) contoured at 3σ as well as in the 2Fo-Fc map (blue) contoured at 2σ…

(b)

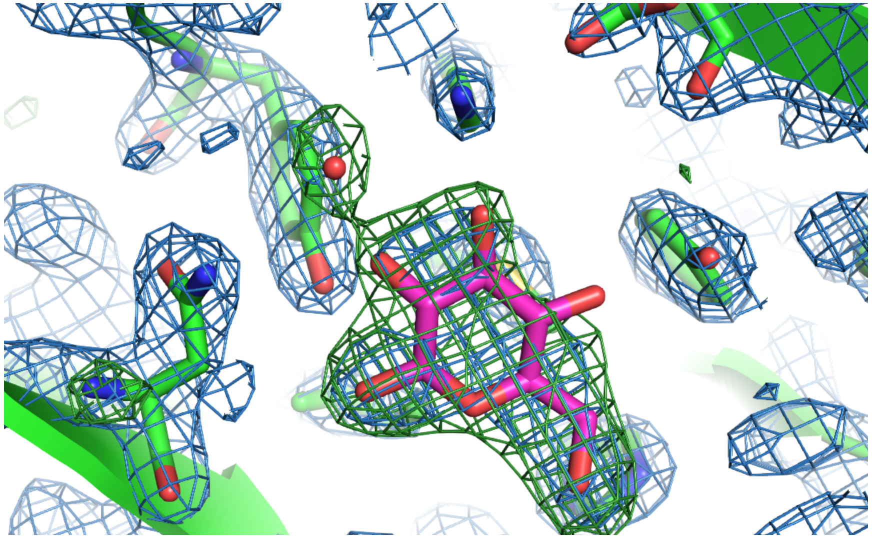

… and its interpretation as a glucose molecule. Here the blue map corresponds to a refined 2Fo-Fc map, contoured at 2σ.

Fig. S7 related to Figure 3

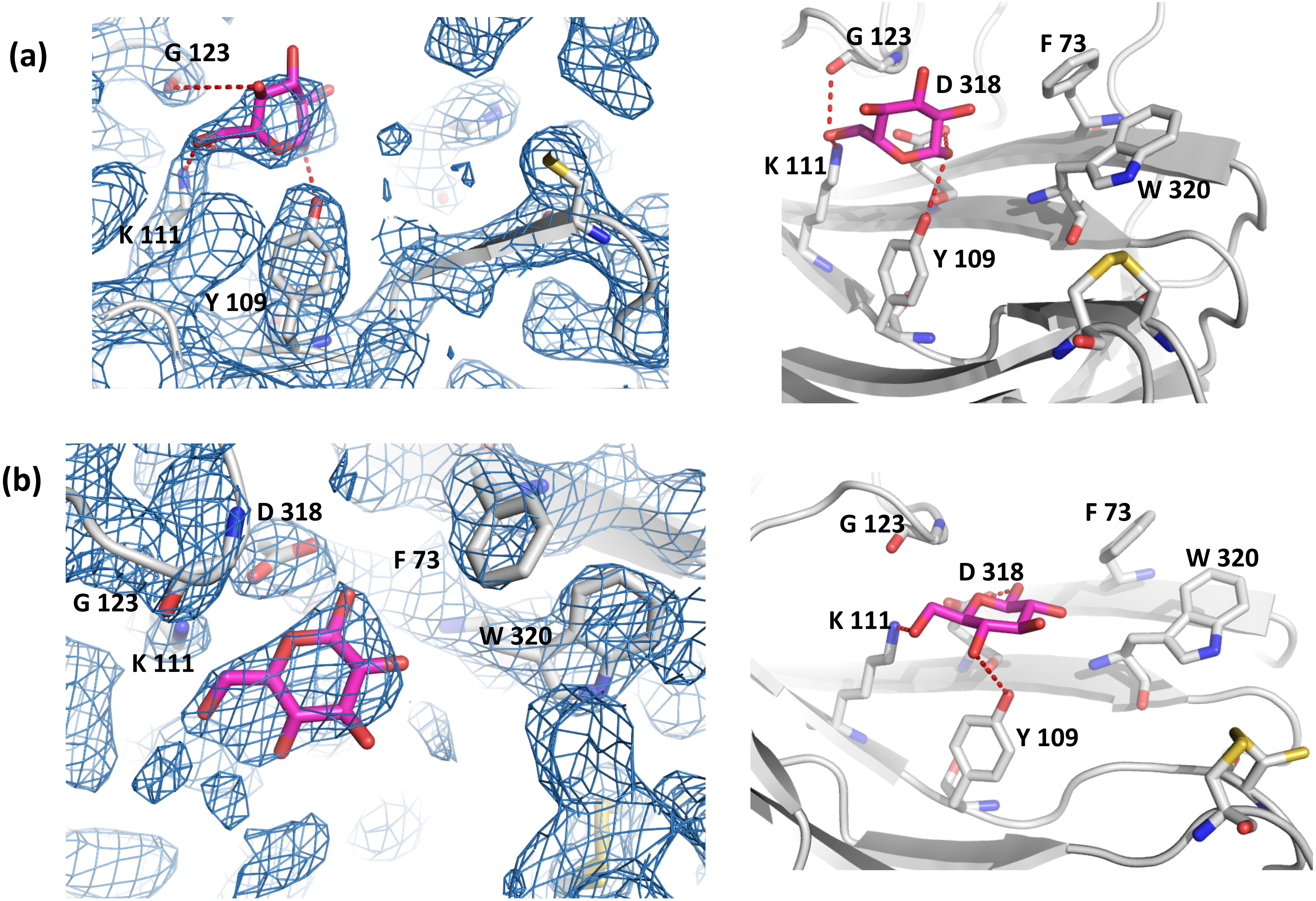

2Fo-Fc electron density maps (2σ) and their associated models for the two Glc positions in TcCRT crystals

Fig. S8, related to figure 3.

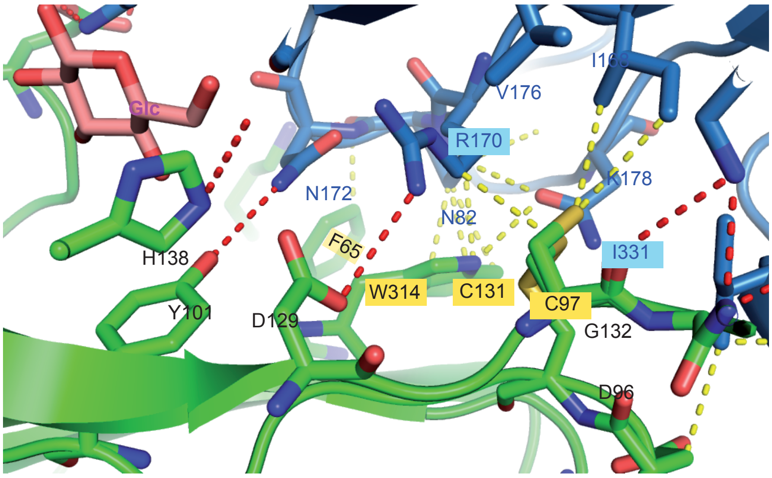

Details of PBS interaction with the closed conformer (blue). Same color code as Fig. 3e. PBS residues are highlighted in yellow. Residues with significant displacement or altered conformation between the open and closed conformations are highlighted in light blue. Some van der Waals contacts are shown with yellow dashed lines.

Figure S9, related to Figure 4 and Table 3

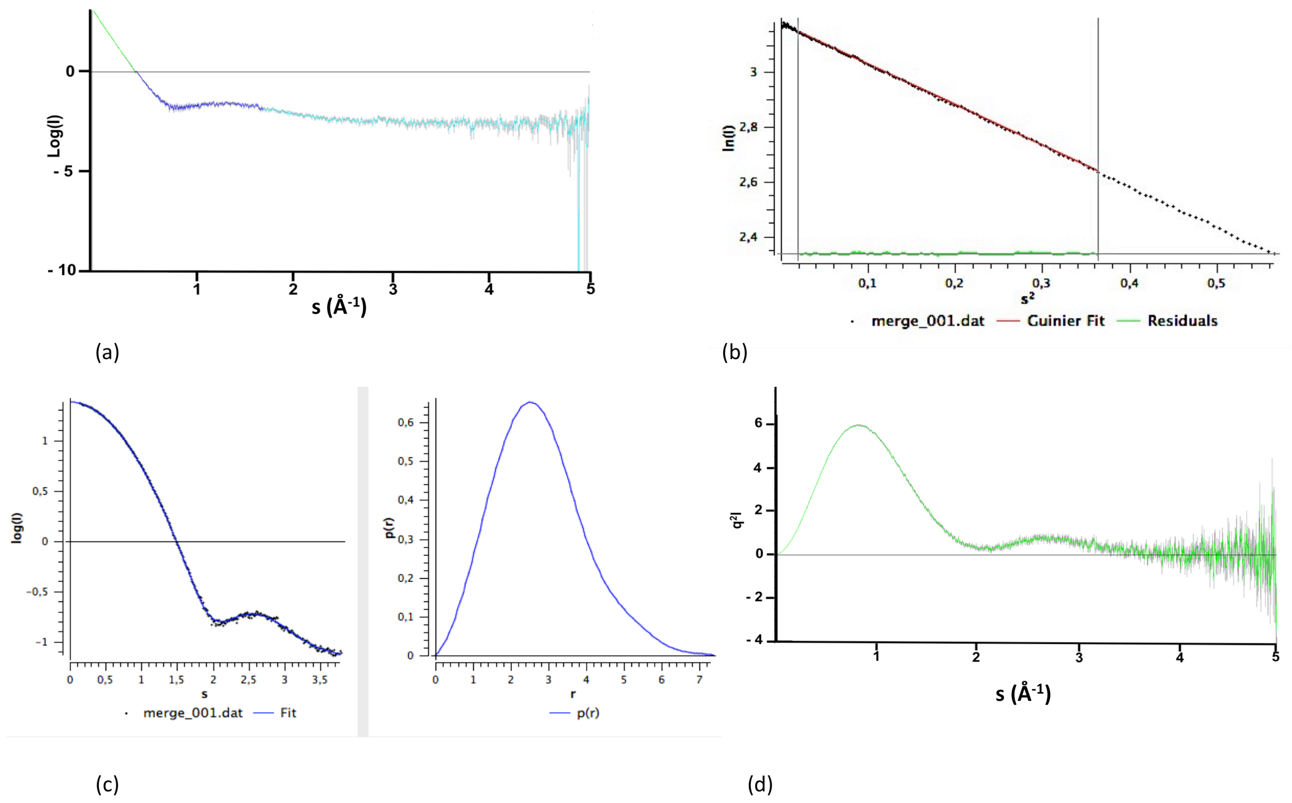

Details of the SAXS analysis of the crystallized TcCRT fragment.

(a). The optimized curve used to calculate structural parameters was obtained by merging the data collected at 1.8 (small q), and 11.15 mg/mL (middle and high q). **(b)** The Guinier plot shows a good fit in the low s^2 region. **(c)** The pair distance distribution function P(r) is characteristic of the globular shape of the crystallized fragment of TcCRT. **(d)** The Kratky plot is typical of a globular protein without any significant flexible region.

Figure S10, related to Figure 4 and Table 3

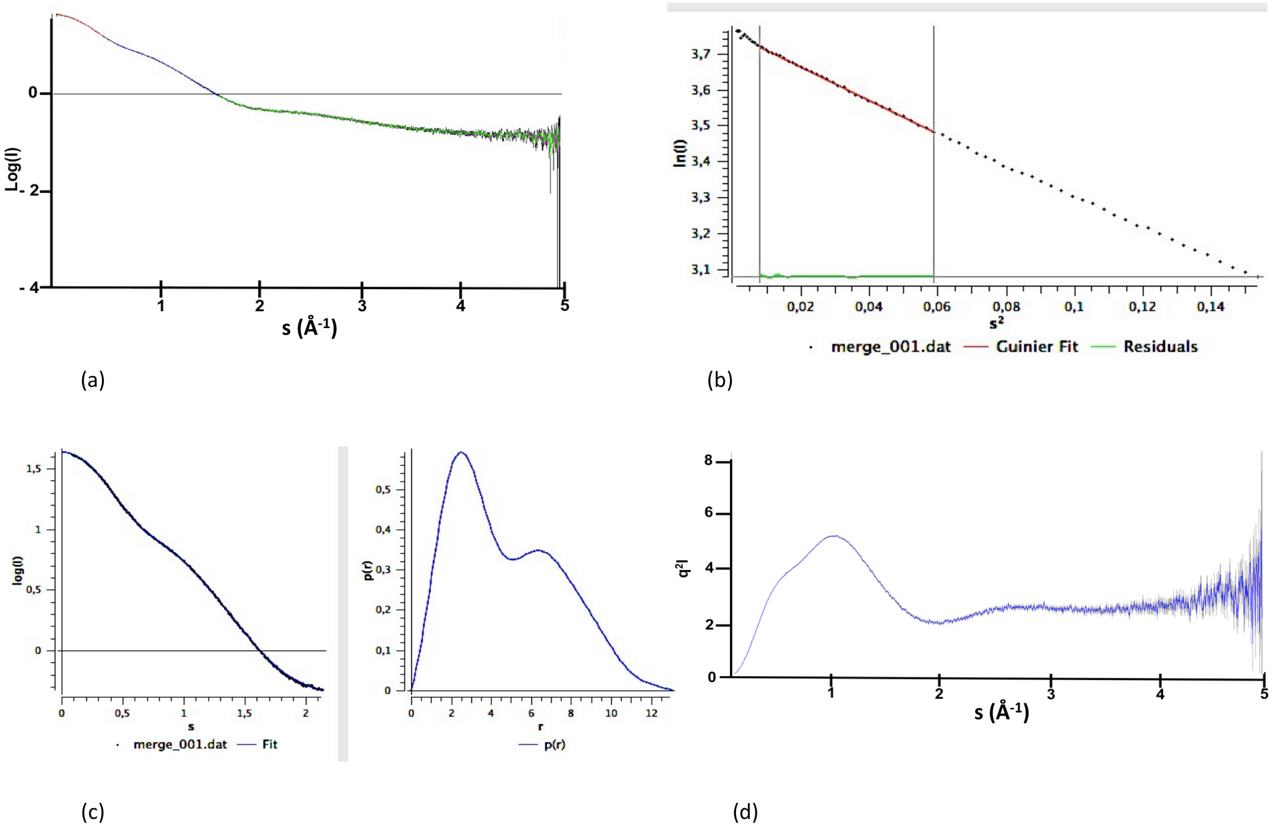

Details of the SAXS analysis of the full-length TcCRT (a). The optimized curve used to calculate structural parameters was obtained by merging the data collected at 1.63 (small q), 7.05 (middle q) and 10.4 mg/ml (high q). **(b)** The Guinier plot shows a good fit in the low s^2 region. **(c)** The pair distance distribution function P(r) is characteristic of the globular domain and the elongated P arm of the full-length CRT. **(d)** The divergence from the baseline of the Krakty Plot for high s values reflects the presence of a flexible domain.
