# Supplementary figures and images for: Structures of parasite calreticulins provide insights into their flexibility and dual carbohydrate/peptide-binding properties
Source: IUCrJ. 2016 Sep 14;3(Pt 6):408–19. doi: 10.1107/S2052252516012847 (PMC5094443; doi:10.1107/S2052252516012847)

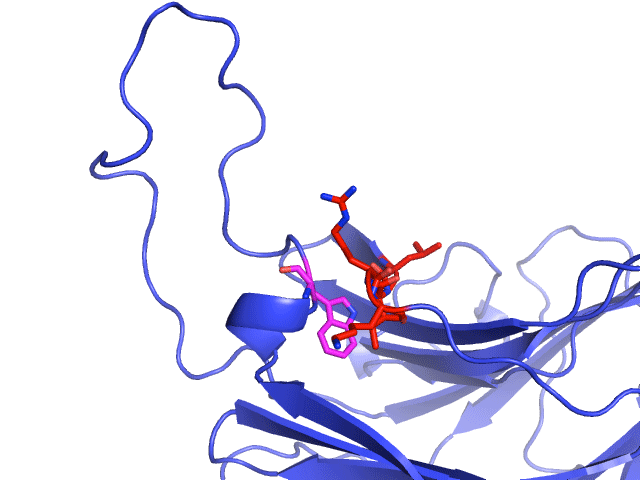

Supplement: Supplementary file 2 [file m-03-00408-sup2.gif]
